# Will PET/MR Imaging Replace PET/CT for Pediatric Applications?

**DOI:** 10.3390/diagnostics15091070

**Published:** 2025-04-23

**Authors:** Gabriele Masselli, Chiara Di Bella

**Affiliations:** Department of Radiological Sciences, Oncology and Pathology, Policlinico Umberto I, Sapienza University of Rome, 00161 Rome, Italy; chiaradibella30@gmail.com

**Keywords:** PET/MR, PET/CT, pediatric, nuclear medicine, imaging

## Abstract

The combination of positron emission tomography (PET) and magnetic resonance imaging (MRI) is a modern, highly advanced diagnostic tool that offers numerous advantages in the treatment and management of some pediatric pathologies. The use of PET/MR in children provides high-resolution images with outstanding tissue characterization, as well as important metabolic and physiological information; it is not only essential for early diagnosis, but also for the assessment and management of oncological, neurological, and cardiovascular diseases. The hybrid PET/MR is a multimodal approach that reduces the need for separate examinations, minimizes radiation exposure, and improves the overall experience for pediatric patients. In addition, PET/MR, by combining functional data, allows for more precise therapeutic planning and monitoring of treatment responses, optimizing clinical interventions especially with regard to staging and follow-up. This review will explore the benefits, weaknesses, and emerging applications of PET/MR in pediatric patients.

## 1. Introduction

Targeted radioactive tracers are used in nuclear medicine techniques to assess organ function and cellular processes, providing valuable insights into pediatric cancer evaluation; key tracers include technetium-99m-labeled bisphosphonate BS and ^18^F-FDG PET [1]. ^18^F-FDG PET is particularly valuable for detecting hypermetabolic malignancies, offering full-body imaging with high sensitivity, though its specificity can be affected by inflammation or infection in lymph nodes. When interpreting PET scans, standardized uptake values (SUVs), a semi-quantitative measure of radiotracer activity, can confirm a metabolic response to therapy by comparing pre- and post-treatment scans. However, SUVs alone cannot reliably differentiate between malignant and benign lesions [1].

The role of magnetic resonance imaging (MRI) continues to advance with enhanced coils, the development of faster scanning methods and improvements in post-processing techniques.

MRI workhorse includes excellent spatial resolution and great soft tissue contrast (leading to superior soft tissue characterization), but also the absence of ionizing radiation and multiplanar capabilities. However, its limitations involve relatively long scan times, limited availability in certain settings, artifacts from physiological motion, suboptimal lung evaluation, and high costs. Additionally, the use of whole-body MRI (WBMRI) for diagnosing, monitoring, and surveilling pediatric cancer patients or those with predispositions has expanded recently due to technological advancements, faster acquisition times, and broader access to MRI [2]. The diagnostic accuracy of MRI is superior to CT, especially in the study of some anatomical districts such as the brain, neck, bone marrow, and pelvis as well as for the study of lymph nodes.

The combination of PET and MRI is a modern, highly advanced diagnostic tool that offers numerous advantages in the treatment and management in pediatric oncologic and non-oncologic processes, in addition to its role in research [3,4,5,6,7,8].

The combination is particularly relevant in pediatric patients due to reduced radiation burden compared with PET/CT, as well as its ability to obtain exquisite functional and anatomic imaging in a single imaging session, thereby reducing the number of anesthesia/sedations [9,10,11,12].

This review will explore the benefits, weaknesses, and emerging applications of PET/MR in pediatric medicine.

## 2. Comparison Between PET/MR Imaging and PET/CT

PET/MR imaging can be performed with a sequential or synchronous system. In a synchronous system, the solid-state PET detectors are located between the body and gradient coils in the 3T MR imaging gantry, which allows for truly synchronous data acquisition [8]. In a sequential system, PET and MRI imaging are performed separately with transportation of the patient between scanners.

A sequential system is technically simpler to implement since most hospitals already possess distinct MRI and PET devices. However, a significant disadvantage is the non-simultaneous nature of the scans, which may lead to misalignment artifacts, extended scan durations, and prolonged sedation needs.

Consequently, a simultaneous PET/MR system is generally more desirable for pediatric neuroimaging (Table 1).

Hybrid PET/MR integrates the distinct advantages of MRI, such as diffusion-weighted imaging, superior soft tissue contrast, dynamic contrast-enhanced imaging and spectroscopy, with the quantitative physiological data offered by PET.

Integrated PET/MR offers cancer staging and follow-up with up to 80% less radiation exposure than PET/CT by using MRI instead of CT for the anatomical colocalization of radiotracer data [3].

The advantages of PET/MR over PET/CT generally include simultaneous acquisition of PET and MRI, overall reduction in radiation absorbed and total examinations performed, it being a 1-stop imaging procedure reduces the need for repeated anesthesia or sedation, thereby shortening the overall scan time compared to conducting the two imaging studies separately [3].

The lower radiation exposure is particularly important for children and young adults who may need repeated follow-up imaging studies over an extended period. Moreover, the pediatric population is most sensitive to ionizing radiation.

In one study in 2020, it reported a dose reduction of 79.6% when comparing PET/MR to PET/CT in 1003 whole-body staging exams [4].

Repeating PET/CTs, especially in pediatric neuro-oncology, results in a considerable cumulative radiation dose and may increase the risk of secondary cancer [13,14,15].

Other advantages of PET/MR are the better characterization and measurement of lesions compared to computer tomography (CT) and the possibility of immediate characterization of incidentalomas [3]. Although MR in most cases can support the diagnosis of incidentaloma, further diagnostic steps, such as biopsy, are often still needed to deepen the diagnosis.

PET/MR allows for a better study of bone marrow lesions and bone metastases and can be an excellent tool in guiding core biopsies [3].

Paramagnetic contrast enhancement (Gadolinium) is not considered necessary at the moment [4], as it is often vicariated by the functional information guaranteed by PET and the information provided by DWI. However, it is advisable to evaluate individual cases, discuss the specific diagnostic question with clinicians, and consider the choice to add contrast sequence if a better characterization of the tissues is necessary in doubtful cases.

Generally, pediatric standard protocol utilizes a 2-point Dixon T1-weighted 3D sequence for attenuation correction and localization in standard planes. For fluid-sensitive imaging during PET bed acquisition, fast sequences such as HASTE and single-shot fast spin-echo are used. Additionally, fluid-sensitive options like the 2-point Dixon T2-weighted or coronal STIR sequences are available. Diffusion-weighted imaging (DWI), with b values of 50, 400, and 800 s/mm^2^, enhances the evaluation of tumors [12].

Moreover, with time-of-flight PET/MR imaging, there is the potential to maintain diagnostic image quality while using only half the FDG dose [5]. Minamimoto et al. conducted a study on twenty-five patients that suggested that TOF PET in PET/MR can reduce various image reconstruction artifacts compared to non-TOF PET/MR, TOF PET/CT, and non-TOF PET/CT [16]. TOF PET works by measuring the difference in arrival times of two coincidence photons, narrowing the emission event’s location, and reducing image noise and voxel cross-dependencies; this results in improved lesion detectability, especially near large hot regions, and reduces errors in normalization, attenuation, and scatter corrections [17,18,19,20]. The current TOF PET/MR system has significantly better timing resolution than PET/CT, leading to better signal-to-noise and contrast-to-noise ratios and, additionally, TOF PET helps reduce metal artifacts, improving lesion detection in areas affected by metallic implants [21,22,23].

While PET/MR hybrid imaging holds significant promise for both diagnosis and follow-up, it faces several limitations and challenges, including limited global availability, high costs, the absence of standardized imaging protocols, the need for frequent sedation in pediatric patients, and longer acquisition times ranging from 60 to 90 min compared to the 30 min typically required for PET/CT [5].

Although MRI often requires prolonged sedation, the use of PET/MR can significantly reduce the need for multiple separate examinations, thus minimizing the frequency of sedation events. The scanning time for PET/MR largely depends on the specific MRI pulse sequences selected [23]. In order for PET/MR to remain competitive with PET/CT, it is ideal for image acquisition to be completed within 30 min but achieving this time frame may only be feasible by avoiding diffusion-weighted imaging (DWI) sequences and substituting coronal STIR sequences with an axial T2-weighted 3-point Dixon technique, which allows for faster imaging without compromising diagnostic quality [23,24,25,26,27,28,29,30].

Due to low proton density in the lungs, MRI struggles with a low signal-to-noise ratio and motion artifacts caused by breathing and cardiac activity.

Turbo inversion recovery magnitude (TIRM) and T1-weighted (T1w) controlled aliasing in parallel imaging results in higher-acceleration (CAIPIRINHA) Dixon with water contrast showing good accuracy when evaluating lung nodules and should be included in the protocol for the evaluation of oncological patients at risk of pulmonary metastatic spread [3,31,32,33,34]. The higher sensitivity of TIRM, rather than T1w, suggests that the fluid-sensitive sequence favors a higher visibility of lesions. Additionally, the sensitivity of ^18^F-FDG-PET/MR hybrid imaging is comparable to that of PET/CT in detecting nodules bigger than 4 mm [3,35].

Looking ahead, advancements in reducing acquisition times, lowering costs, increasing global availability, and refining protocols will enhance the accuracy of lung diagnostics with PET/MR.

## 3. Clinical Applications

### 3.1. Hematology

#### Lymphoma

Lymphomas are primarily classified into two main groups: Non-Hodgkin lymphoma (NHL), which is staged using the Pediatric Non-Hodgkin lymphoma Staging System, and Hodgkin lymphoma (HL), which follows the Ann Arbor staging system. In both adult and pediatric oncology, ^18^F-FDG-PET plays a crucial role in the study, staging, and therapeutic evaluation of lymphomas [12]. ^18^F-FDG PET/MR and PET/CT have demonstrated nearly equivalent performance in identifying lymphomas, determining Ann Arbor classification, and evaluating post-treatment response in pediatric patients [3]. Additionally, ^18^F-FDG-PET/MR is superior to core biopsy in assessing bone marrow involvement, allowing biopsy to be avoided if the PET/MR scan shows no involvement [3].

In addition, PET/MR provides exceptional accuracy, particularly in studying conglomerated lymph node masses and measuring lymph nodes. This is further enhanced by T2-weighted sequences and the reliable confirmation of diffusion-weighted imaging (DWI), which shows high restriction [4]. PET/MR also serves as a valuable tool in evaluating post-transplant lymphoproliferative disorders [12].

A further advantage of PET/MR is represented by the possibility of avoiding the use of contrast medium, since some studies have shown that the use of contrast agents in PET/MR examinations with ^18^F-FDG has no beneficial effects in the primary and follow-up staging of children with lymphoma [36]. Therefore, the possibility of using PET/MR protocols with ^18^F-FDG without contrast medium avoids the risk of adverse effects and reduces the time and costs of the examination [36,37].

### 3.2. Musculoskeletal

#### Bone Tumor

The most common primary bone cancers in children are osteosarcoma and Ewing’s sarcoma. MRI is regarded as the standard approach for local diagnosis and staging. For both types of neoplasms, there has been a shift from bone scintigraphy to ^18^F-FDG-PET for staging and therapy response assessment when available [4]. MRI plays a pivotal role in determining tumor size and invasiveness; it is highly effective in differentiating non-viable soft tissue from active tumors, delineating the extent of local tumor invasion versus peripheral inflammatory changes, and evaluating bone marrow involvement. However, when integrated with PET, the functional and metabolic data from PET further enriches this analysis. This combination enables a more comprehensive assessment of tissue viability and tumor behavior, enhancing both diagnostic accuracy and treatment planning. The exceptional soft-tissue contrast provided by PET/MR is particularly beneficial in musculoskeletal imaging, offering significant advantages in diagnosing bone marrow abnormalities, soft tissue conditions, and ligament or tendon pathologies. In these cases, hybrid PET/MR imaging allows for both local and whole-body evaluation in a single session. It is especially valuable in preoperative planning for musculoskeletal tumors, as it allows for precise identification of neurovascular structures that must be preserved during resection. Diffusion-weighted imaging (DWI) is particularly useful for assessing therapy response, offering more reliable results than dimensional changes or post-contrast enhancement alone. It is also important to note that patients with osteogenic and soft-tissue sarcomas who respond to chemotherapy show an increase in tumor apparent diffusion coefficient (ADC) values [3]. The most common sites of metastasis are the lymph nodes and lungs. Lymph node involvement can be easily detected with DWI, while chest CT is still needed to assess pulmonary lesions. MRI is the imaging modality of choice for diagnosis and local staging, while combined ^18^F-FDG PET/MR enhances staging accuracy and response assessment by providing both metabolic and structural details. PET is especially valuable for distinguishing perilesional edema from tumor infiltration and identifying tumor thrombi, as edema exhibits low or absent ^18^F-FDG uptake, whereas tumor thrombi demonstrate high uptake due to the presence of metabolically active cancer cells; tumor thrombi are often linked to visible masses and may invade adjacent structures [22].

Ewing sarcoma usually originates in bone structures but can also develop in soft tissues, with potential metastases to the lungs and bone marrow [4]. PET/MR diagnosis enables simultaneous evaluation of therapy response, pre-surgical planning, and differentiation between edema and tumor tissue [4].

In osteosarcoma, the soft-tissue component is typically hyperintense on T2-weighted MRI with high ^18^F-FDG uptake, while the bone-forming component appears hypointense on T2-weighted MRI with low uptake [22]. PET/MR is also an important and useful tool for guiding biopsies in Ewing sarcoma because of its ability to identify the hypermetabolic area, but it is also very important to evaluate chemotherapy response that can be considered good when there is more than a 30% reduction of the ^18^F-FDG uptake [22].

MRI is highly effective for soft tissue imaging, offering excellent contrast and detailed characterization, but it does have limitations when it comes to distinguishing between viable tumor tissue and non-viable scar tissue or treatment-induced inflammatory changes. This is where the combination of PET and MRI becomes particularly valuable. By integrating functional information from PET with the detailed anatomical imaging of MRI, PET/MR provides a more comprehensive diagnostic picture. This fusion of data allows for a more accurate assessment, enabling better differentiation between viable tumor and other tissue changes in a single examination.

Eiber et al. conducted a study on 119 patients with bone metastases undergoing PET/CT and subsequent PET/MR, and they found superior results for PET/MR in anatomical delineation and allocation using a T1-weighted turbo spin-echo sequence [32].

### 3.3. Neuroendocrine System

#### Neuroendocrine Tumors

Pheochromocytomas and paragangliomas are very rare neuroendocrine tumors in children [20]. Traditionally, imaging for these tumors relied on ^111^In-octreotide, but both ^68^Ga-DOTA peptides and ^18^F-fluorodihydroxyphenyl-l-alanine (^18^F-DOPA) PET/computed tomography (CT) have recently become the preferred modalities due to their superior sensitivity and specificity [20,21]. However, FDG PET still plays a role in managing certain neuroendocrine tumor subtypes, particularly those with more aggressive behavior.

Although neuroendocrine tumors are uncommon in pediatric patients, the identification of an increasing number of cancer predisposition syndromes (CPS)—which include neuroendocrine neoplasms—has led to a growing demand for ⁶⁸Ga-DOTATATE PET/CT and PET/MR [21] (Table 2). These advanced imaging techniques provide more accurate tumor localization and characterization, improving both diagnostic precision and treatment planning.

### 3.4. Systemic Diseases

#### Neurofibromatosis Type I

Neurofibromatosis Type I (NF1), or von Recklingahausen disease, is a multisystemic disorder caused by a genetic mutation that causes (mainly benign) tumors called neurofibromas along the nervous system that can grow in any part of the body. There is the possibility that neurofibromas degenerate into malignant peripheral nerve sheath tumors (MPNSTs).

In these patients, MRI allows a detailed assessment of neurofibromas in both the central and peripheral nervous systems using the STIR, FLAIR, and DWI sequences (showing proton restriction in benign and malignant lesions), while ^18^F-FDG PET provides valuable information on the metabolic activity of MPNSTs, which show a high absorption of FDG with SUV values ranging from 2.5 to 6. The combination of these two elements in a single hybrid system allows a clear increase in diagnostic accuracy [3,4]. Compared to PET/CT, this approach offers improved lesion characterization and a better understanding of their transformation into MPNSTs, while also reducing radiation exposure and allowing for the identification and characterization of necrosis foci [4,28].

### 3.5. Langerhans Cell Histiocytosis

Langerhans cell histiocytosis (LCH) is a disorder of dendritic cells, which can be either multisystemic or involves focal areas. Bone is affected in 70% of cases [4]. Initially, the monitoring of the disease was primarily conducted through skeletal radiography; later, scintigraphy and MRI were introduced for a more detailed diagnostic evaluation [4]. The incorporation of ^18^F-FDG-PET alongside MRI has been shown to reduce the rate of false positives [4].

### 3.6. Inection and Inflammatory Disease

Both ^18^F-FDG-PET and MRI have proven to be highly accurate techniques for diagnosing and characterizing infectious and inflammatory processes such as gastro-intestinal inflammatory diseases (Crohn’s disease or ulcerative colitis), sarcoidosis, rheumatoid arthritis, psoriatic arthritis, autoimmune disorders (e.g., Sjogren’s syndrome), vasculitis, and systemic lupus erythematosus (LES) [6]. These infectious and inflammatory conditions can occur independently or coexist in pediatric cancer patients, complicating both their clinical and radiological presentations. Hybrid PET/MR imaging can be particularly beneficial in these cases, offering enhanced characterization due to the hypermetabolism observed in conditions like appendicitis, otitis, pneumonia, and odontogenic infections [3]. Therefore, PET/MR is crucial in preventing the misinterpretation of acute inflammatory processes as neoplastic conditions, which could lead to unnecessary changes in treatment.

Rodrigo et al. examined the brain metabolic activity of a cohort of systemic lupus erythematosus patients with neuropsychiatric symptoms (j-NPSLE) who underwent ^18^F-FDG PET with the aim of searching for foci of hyperactivity (inflammation) or hypoactivity (ischemia), since brain MRI is generally negative or abnormalities are nonspecific in more than 50% of patients with NPSLE [38]. The analysis showed a diffuse bilateral cortico-subcortical hypermetabolism, which was predominantly subcortical and mostly thalamic; this finding was also present in patients with negative MRI and normal neurological examination [37]. This demonstrates the pivotal role that ^18^F-FDG PET can have in improving the diagnostic accuracy of this category of patients and we believe that hybrid ^18^F-FDG PET/MR imaging can further increase the diagnostic power by offering together the advantages of detailed anatomical study and brain metabolic activity in a single examination that offers better visualization of brain tissues and a lower radiation dose than PET/CT.

Hybrid imaging also may be useful to detect early inflammation in Crohn’s disease (CD), improving treatment and reducing costs. Combining PET and MRI offers advantages over PET/CT, SPECT/CT, or MRI alone, as simultaneous PET/MR acquisition allows better alignment of dynamic processes, particularly in the abdomen [24,25,26,27]. MRI enhances PET data analysis, especially in pharmacokinetic modeling, by providing perfusion and blood flow information. Integrating PET with anatomical and functional MRI (e.g., DWI, spectroscopy) as well as with advanced contrast agents improves lesion characterization and non-invasive histological assessment [25].

Despite the wide range of possibilities, results from PET/MR in children with chronic inflammatory bowel disease are still mixed. Dalby et al. conducted a study on patients aged 8–17 with suspected IBD showing that the accuracy of PET/MR in detecting intestinal inflammation was not clearly superior to PET and MRI alone; PET/MR did not detect clinical response or state of biochemical response nor did it show the same diagnostic accuracy demonstrated in adult patients with IBD [28].

### 3.7. Neurology

PET and MRI offer supplementary insights into the analysis of the human brain. Concurrent PET/MR data collection enables the alignment of spatial and temporal signals, unlocking possibilities that cannot be achieved with separate devices [30].

CNS tumors are the most common solid tumors in children and young adults, accounting for over 25% of all cancers, with astrocytoma, medulloblastoma, and ependymoma being the most frequent types [7]. Imaging, particularly MRI, plays a vital role in diagnosis, treatment planning, and monitoring, but challenges arise due to treatment-related changes and phenomena like pseudo-progression and pseudo-response, necessitating the use of advanced imaging techniques and PET for improved diagnostic accuracy and better patient outcomes; however, in pediatric neuro-oncology, hybrid PET/MR is not commonly used in clinical practice and should be regarded as a research tool [7].

PET is generally useful for distinguishing high-grade from low-grade brain tumors, predicting prognosis, evaluating tumor extent to optimize resection and radiotherapy, identifying the most appropriate biopsy site, and detecting relapses. MRI, on the other hand, is crucial for assessing tumor characteristics such as enhancement, necrosis, and extension on pre- and post-contrast T1 sequences, as well as peritumoral edema (vasogenic and infiltrative), blood products, calcifications, radiation-induced chronic micro-hemorrhages, and non-enhancing tumors on T2 sequences. Additionally, MRI plays an important role in whole-body evaluation for locoregional staging and detecting distant metastases [8,9]. In pediatric neuroimaging, synchronous PET/MR is preferred to avoid artifacts and minimize sedation time compared to asynchronous PET/MR [8].

While complete resection remains the primary treatment for most tumors, the extent of resection significantly influences prognosis, with postoperative MRI being the standard method for assessing resection within the first 0–72 h [10]. However, postoperative signal changes can arise from tissue responses to surgery, such as bleeding, edema, or artifacts caused by metal implants, complicating interpretation. Postoperative MRI imaging may be equivocal or miss small residual lesions. In this setting, PET can be used to evaluate tumor recurrence and detect transformation to a higher tumor grade. MRI imaging allows DWI and FLAIR sequences, which are valuable in brain tumor assessment, as well as the whole-body evaluation of metastatic disease. The addition of PET imaging within the first 72 h after surgery can help distinguish residual tumor from post-surgical changes [10]. Although the use of ^18^F-FDG is limited by physiological uptake in gray matter, amino acid analogues like 11C-methionine, ^18^F-fluoro-phenylalanine, and 18-Fluoroethyl-L-tyrosine (^18^F-FET) offer superior accuracy in delineating tumors from normal brain tissue. Residual PET tracer uptake may indicate shorter survival in high-grade gliomas, though significant absorption of these analogues can also occur in non-neoplastic or acute inflammatory lesions [10]. PET with ^18^F-FET combined with MRI has been shown to identify lesions not visible on MRI alone, directly impacting surgical decisions and prognosis in pediatric patients [10].

An interesting neurological non-oncology use of PET/MR is based on the study of epilepsy, particularly on the presurgical epileptogenic foci, because this hybrid method allows a fine evaluation of cortical dysplasia or temporal mesial sclerosis [16]. In particular, some results showed that the sensitivity of ^18^F-FDG PET for temporal lesion localization was reported as 72.2% compared to 82.6% for MRI imaging [17].

Beyond its role in localization, ^18^F-FDG PET plays a crucial part in defining surgical margins for non-lesional temporal lobe epilepsy (TLE). Studies have shown that the extent of resection of the hypometabolic region identified in preoperative ^18^F-FDG PET scans is a strong predictor of postsurgical outcomes [29]. Notably, ^18^F-FDG PET detects hypometabolism in most patients with non-lesional TLE, even in the absence of hippocampal atrophy, further underscoring its value in surgical planning and prognosis [16,18].

In an evaluation comparing brain uptake of ^18^F-FDG in PET/MR and PET/CT, Sager et al. found that FDG absorption is symmetrical in both imaging modes in the left and right brain regions and that there are similar SUV values in both the ^18^F-FDG PET/MRI and PET/CT images of the cortical and subcortical regions of the brain except from the SUVmax value of the left-sided parietal lobe [29].

Caligiuri et al. performed a study on patients with Huntington’s disease in order to evaluate with ^18^F-FDG PET/MR the difference between the brains of children with pediatric-onset Huntington’s disease (POHD) and patients with adult-onset HD (AOHD), demonstrating that POHD presents a much more severe striatal volume loss than AOHD and that only in POHD is there an alteration of glucose metabolism in several areas of the cerebral cortex and in the thalamus; this not only highlights important differences between the two forms of HD but also highlights that hybrid ^18^F-FDG-PET/MR is a valuable tool for the combined study of volumetric and metabolic changes in the same individual and for monitoring POHD [36].

### 3.8. Cardiology

Cardiac PET/MR in pediatric patients is a tool destined for great development and evolution. Kwatra et al. suggested several application perspectives, such as the study of morpho-functional variations in patients with congenital anatomical abnormalities or congenital heart disease, as well as myocardial perfusion assessment for post-evaluation heart transplant or Kawasaki disease [16].

In sarcoidosis, ^18^F-FDG PET and MRI with gadolinium are utilized to assess cardiac involvement, determine risk levels, and monitor response to treatment [16].

In oncology, cardiac MRI is the primary modality for assessing the heart’s tumor morphology; the initial step involves comprehensive imaging of the entire heart in axial or four-chamber view stacks to accurately locate the tumor for further detailed evaluation [19]. The MRI protocol includes functional imaging, T1-weighted sequences with and without fat suppression, T2-weighted imaging, first-pass perfusion, and LGE imaging; combining PET with MRI provides additional metabolic insights, enhancing tissue characterization with optimal alignment for precise assessment [19].

Cardiac MRI has demonstrated strong diagnostic performance in the detection of acute myocarditis, yet it remains suboptimal in identifying chronic myocarditis, with a reported diagnostic limitation. T2-weighted imaging, a key tool for distinguishing between acute and chronic myocardial inflammation, is particularly vulnerable to motion artifact, often resulting in inconsistent image quality, especially in patients experiencing arrhythmias or other motion-related disturbances. FDG-PET offers the distinct advantage of directly visualizing metabolic activity within inflammatory infiltrates, enabling the detection of myocarditis in both acute and chronic stages [32].

Future development of hybrid PET/MR could lead to more precise and comprehensive diagnostic capabilities, fostering the development of personalized treatment strategies, with the potential of evolving technology to improve sensitivity, reduce motion artifacts, and enhance overall diagnostic accuracy, which could significantly refine our approach to managing myocarditis, ultimately improving patient outcomes and informing future therapeutic advancements.

### 3.9. PET MRI: Limitations and Future Research

This integrated approach with the use of several tracers, depending on the clinical scenario, is particularly useful for assessing responses to cancer immunotherapy, as it incorporates established criteria such as RECIST and immune-related RECIST, which are widely used in immunotherapy trials.

A major challenge in evaluating immunotherapy is distinguishing true tumor progression from pseudo-progression, a phenomenon characterized by transient tumor swelling, MRI contrast enhancement, and elevated ^18^F-FDG metabolic activity due to immune cell activation within the tumor.

In contrast to CT, MRI offers superb soft tissue contrast and additional functional tissue characterization by means of the multiple available MR-sequences, such as diffusion-weighted (DWI) and dynamic contrast-enhanced (DCE) imaging.

DCE and DWI sequences have been shown to distinguish pseudo-progression from real progression in different tumor entities and brain metastatic disease under ICIs [39,40].

The combination of whole-body analysis of glucose metabolism with mpMRI-derived data offered by integrated ^18^F-FDG PET/MR may significantly aid in the early prediction of treatment response to immunotherapy.

Therefore, advanced PET/MR techniques may play a crucial role in differentiating pseudo-progression from true tumor growth, thereby improving treatment assessment and decision making.

In addition to oncology, PET/MR offers significant benefits in neurology, systemic diseases, and inflammatory conditions by reducing radiation exposure, improving diagnostic accuracy, and optimizing clinical interventions. Its ability to simultaneously acquire anatomical and functional data makes it particularly valuable for treatment planning and disease monitoring, as demonstrated in conditions such as lymphomas, sarcomas, and neurofibromatosis. Despite these advantages, PET/MR still faces limitations, including limited availability, high cost, and prolonged scan times, particularly in the evaluation of small lung nodules. Ongoing advances aim to overcome these barriers, ultimately striving to make PET/MR a more accessible, efficient, and widely implemented imaging modality for pediatric patients worldwide.

Future studies will require further knowledge of the actual diagnostic and follow-up benefits of PET/MR for pediatric patients through targeted studies, multicenter studies, and high-volume studies. A significant engineering evolution of PET/MR machines and software will be necessary, as well as the development of optimal and short study protocols.

## 4. Conclusions

In conclusion, hybrid PET/MR imaging represents a highly promising advance in pediatric medicine, providing high-resolution imaging with superior tissue characterization than PET/CT while simultaneously providing critical metabolic and physiological information.

## Figures and Tables

**Table 1:** Advantages of MRI compared to CT.

Aspect	MRI	CT
Soft Tissue Contrast	Superior soft tissue contrast; ideal for brain, spine, joints, and organs	Lower soft tissue resolution
Radiation Exposure	No ionizing radiation (safe for repeated use and certain populations)	Uses ionizing radiation
Detail in Brain Imaging	Excellent detail; preferred for tumors, multiple sclerosis, stroke	Limited soft tissue detail
Functional Imaging	Can perform fMRI for brain activity and spectroscopy	Functional imaging limited or not possible
Contrast Agents	Uses gadolinium (lower risk of allergy compared to iodine in CT)	Iodinated contrast can cause more allergic reactions
Bone Artifact Interference	Less interference from bones in imaging soft tissues	Bone can obscure soft tissue structures
Chronic Disease Monitoring	Better for long-term follow-up due to lack of radiation	Not ideal for frequent imaging

**Table 2:** PET/MR imaging radiopharmaceutical, as well as oncological and non-oncological applications.

PET Radiopharmaceutical	Clinical Application	Pathology
^18^F-FDG	Oncology	-Lymphoma -Primary bone tumors -CNS tumors -Neuroendocrine tumors -NF1
^18^F-FDG	Nononcologic	-BD -Metabolic cardiac study -Vasculitis -Epilepsy -LES -Arthritis -Sarcoidosis -Sjogren’s syndrome -LCH
^68^Ga-DOTATATE	Oncology	-Neuroendocrine tumors -Neuroblastoma
^18^F-DOPA	Oncology	-Neuroendocrine tumors -Primary bone tumors -Neuroblastoma
^18^F-FET	Neuro-oncology	-Brain gliomas.

## Data Availability

Not applicable.
